# Circular Savannah Medical College

**Published:** 1857-05

**Authors:** 


### "CIRCULAR OF SAVANNAH MEDICAL COLLEGE."

A friend has placed upon our table, at a late hour before closing up the editorial matter for this Journal, the circular of the Savannah Medical College, which reports, after a number of years, twenty-five students in attendance during the last course of lectures, and announces itself authoritatively and uncompromisingly opposed to teaching medicine in the summer, and with *nothing to lose*, comes up to the help of the Nashville School, hoping, by possibility, something *may be gained* for themselves in the contest.

We think this whole *scheme* is understood by us, and though most signally defeated in the accomplishment of what was intended as the initiatory step in this *combined* movement at the late meeting of the Medical Society of the State of Georgia, they yet hope to secure the accomplishment of their object, through what is supposed to be the all controlling influence of those to whom we have good reason to believe, they are committed.

But we have already bestowed more attention upon this circular than we had intended, for indeed, our principal feeling in the matter has been a rather excessive amusement, and as we are in the way of using Esop, we will quote him again, as more appropriate to the subject than any thing that we could possibly conceive—"A gnat that had been buzzing about the head of a bull, at length settling himself down upon his horn, begged his pardon for incommoding him; but if," says he, "my weight at all inconveniences you, pray say so, and I will be off in a moment." "Oh, never trouble your head about that," says the bull, "for 'tis all one to me, whether you go or stay; and to say the truth, I did not know you were there."

